# Statistics

**Published:** 1847-01

**Authors:** 


### STATISTICS.

*Deaths in Charleston during the Months of July and August,* 1846.

*July.*—Deaths 50. (Adults, 24; children 26.) By cancer, 1; cholera infantum, 1; consumption, 6; convulsions, 3; croup, 1; debility, 1; diarrhœa, 7; dropsy, 3; dropsy of chest, 1; drowned, 3; dysentery, 1; fever catarrhal, 1; fever congestive, 1; fever country, 1; fever scarlet, 1; hypertrophy of the heart, 1; intemperance, 1; old age, 3; pneumonia, 1; teething, 8; trismus nascentium, 2; ulceration of pylorus, 1; unknown, 1.

*August.*—Deaths 56. (Adults, 30; children, 26.) By bronchitis, 1; cerebritis, 1; cholera infantum, 3; consumption, 8; convulsions, 4; croup, 1; debility, 2; diarrhœa, 3; dropsy, 4; fever country, 1; fever scarlet, 2; fever typhus, 1; gangrene, 1; gastritis, 1; hemorrhage, 1; hepatitis, 1; hooping cough, 1; hydrocephalus, 1; marasmus, 1; old age, 6; sore throat, 1; teething, 5; tetanus, 2; trismus nascentium, 2; unknown, 2.

### *Statistics of the Losses Armies Sustain.*

(Dub. Quart. Journ., August, 1846, p. 164.)—Among the 80,000 men forming the French army in Algeria, in 1842, the deaths were 69 in 1,000 effective men; and 60, in 1843.

| | | | | | | | |
|---|---|---|---|---|---|---|---|
| In | 1840 | there | was | 1 | death in | 6 | patients. |
| " | 1841 | " | " | 1 | " | " 9 | " |
| " | 1842 | " | " | 1 | " | 13 | " |
| " | 1843 | " | " | 1 | " | 23 | " |
| " | 1844 | " | " | 1 | " | 32 | " |

This shows that a marked improvement in the sanitory condition of the French colony has taken place.

### *Comparison between Deaths by Disease and in Battle.*

The French army destined for Egypt, from the time of its departure from France, to the end of the year 1804, lost as follows:

| | | | |
|---|---|---|---|
| Killed in battle, | 3614 | Killed by accidents, | 290 |
| Died from wounds, | 854 | Died from disease, | 4,157 |
| | | Total, | 8,915 |

In the French expedition to the Morea, among 17,000men, during seven months, from 1st Sept. 1828, to 1st April 1829,

840 men died by disease alone, which would give an annual proportion of 84.6 in 1,000.

In the official documents of the British army, published by the Inspector General H. Marshall, the losses of the British army in Spain, for a period of 41 months, from January 1811 to May 1814, in a force of 61,511, were 24,930 deaths by disease, and 8,889 in battle, being for the former 118.6 in 1,000, and for the latter 42.4. Soldiers absent from their corps on account of disease 225 in 1,000, reducing the army to one-fourth of its effective strength; 66 officers were killed in every 1,000 in battle, and 37 died by disease.

In the four battles of Talavera, Salamanca, Vittoria and Waterloo, there were 39 officers killed in each 1,000, and of soldiers and non-commissioned officers 31.1, in the same number. In the British navy from June 1780 to April 1783, the deaths from various diseases were 3,230; killed, 640; and died of wounds, 500. Thus showing, that in the navy as well as in the army, the deaths by *disease* greatly exceed those by *warfare.*

### *Midwifery Statistics.*

(Lond. Lancet, Aug. 1846, p. 191.)—A reviewer in the March number of the Archives Generales, gives the following general results of midwifery statistical tables recently published in the Italian and English Journals. In 47,116 labors, twins occurred 446 times (9 4-10 per thousand) and triplets four times (1 in 10,000.) There were 40,233 head presentations, (969 per thousand) of which 40.046 were vertex, and 187 face. There were 1,065 breech or footing presentations, (27 per thousand) and 154 transverse ones (4 per thousand)—of these labors 46,632 terminated naturally (989 per thousand) and 484 (11 per thousand) artificially, viz: 221 by means of forceps; 89 by craniotomy; 54 by turning, and 20 by vaginal or uterine hysteriotomy.—*South Med. Jour.*

